# The Treatment of Pott’s Disease by the Plaster-of-Paris Jacket

**Published:** 1880-06

**Authors:** 


### THE TREATMENT OF POTT'S DISEASE BY THE PLASTER-OF-PARIS JACKET.

In the Boston *Medical and Surgical Journal*, of May 13, Dr. Bradford, in an interesting communication, reaches the following conclusions:

1. Plaster jackets are efficient in Pott's disease, when caries is below the level of the middle of the scapula.

2. The efficiency is not due to fixation alone, nor to extension in the proper sense of the term, but to fixation in an improved position. This improved position is usually obtained by suspension, but also in many cases by recumbency.

3. The treatment by plaster jacket requires care in the application of the bandage. A poor plaster jacket will do harm, deceiving patient and physician.

